# Interrelationships between molecular subtype, anatomical location, and extent of resection in diffuse glioma: a systematic review and meta-analysis

**DOI:** 10.1093/noajnl/vdz032

**Published:** 2019-10-01

**Authors:** Beverly I De Leeuw, Kirsten M Van Baarsen, Tom J Snijders, Pierre A J T Robe

**Affiliations:** 1 Department of Neurology and Neurosurgery, Brain Center, University Medical Center Utrecht, Utrecht, The Netherlands; 2 Department of Neurosurgery, Alder Hey Children’s NHS Foundation Trust, Liverpool, UK

**Keywords:** extent of resection, glioma, location, molecular markers, WHO 2016 Classification

## Abstract

**Background:**

The introduction of the 2016 WHO Classification of Tumors of the Central Nervous System has resulted in tumor groupings with improved prognostic value for diffuse glioma patients. Molecular subtype, primarily based on IDH-mutational status and 1p/19q-status, is a strong predictor of survival. It is unclear to what extent this finding may be mediated by differences in anatomical location and surgical resectability among molecular subgroups. Our aim was to elucidate possible correlations between (1) molecular subtype and anatomical location and (2) molecular subtype and extent of resection.

**Methods:**

We performed a systematic review of literature searching for studies on molecular subtype in relation to anatomical location and extent of resection. Only original data concerning adult participants suffering from cerebral diffuse glioma were included. Studies adopting similar outcomes measures were included in our meta-analysis.

**Results:**

In the systematic analysis for research questions 1 and 2, totals of 20 and 9 studies were included, respectively. Study findings demonstrated that IDH-mutant tumors were significantly more frequently located in the frontal lobe and less often in the temporal lobe compared with IDH-wildtype gliomas. Within the IDH-mutant group, 1p/19q-codeleted tumors were associated with more frequent frontal and less frequent temporal localization compared with 1p/19q-intact tumors. In IDH-mutant gliomas, greater extent of resection was achieved than in IDH-wildtype tumors.

**Conclusions:**

Genetic profile of diffuse cerebral glioma influences their anatomical location and seems to affect tumor resectability.

**Key Points**

1. Extent of resection and molecular markers are prognostic factors in diffuse glioma.
2. Molecular subtype of glioma affects tumor location and extent of resection.

**Importance of the Study**

Extent of resection is a well-established prognostic factor in diffuse gliomas of all grades. With the introduction of the WHO 2016 classification of central nervous system tumors, the “layered” histological and molecular diagnosis of gliomas has become the new standard. In this systematic review, we demonstrate that a glioma’s molecular subtype affects tumor location and—to a lesser extent—extent of resection. Our findings underscore that the prognostic value of extent of resection cannot be studied fully without incorporating molecular subtype and location. Clinically, our analysis suggests that further research is needed to develop refined neurosurgical guidelines for diffuse glioma, which are stratified by molecular subgroup. Such research and guidelines would further launch oncological neurosurgery into the era of precision medicine.

In 2016, the World Health Organization (WHO) Classification of Tumors of the Central Nervous System underwent major revision. For the first time, molecular characteristics are incorporated in the classification of brain tumors. This allows for more accurate, “layered” diagnosis, improved patient management, and more accurate estimation of prognosis and likelihood of treatment response.^1^ Based on their genetic profile, astrocytomas (IDH-mutant, 1p/19q-intact) and oligodendrogliomas (IDH mutant, 1p/19q-codeleted) are now more homogeneously defined.^2^ IDH-wildtype tumors are either classified as diffuse astrocytoma or glioblastoma, depending on their histological grade.

Since the introduction of the revised classification, multiple studies have investigated outcomes of patients with diffuse gliomas and consistent conclusions are drawn: genetic subtype as outlined in the 2016 WHO classification is a stronger prognostic marker for survival than the earlier histopathological categorization.^3–8^ For this reason, studies based on the WHO 2007 criteria should be reevaluated in light of the new classification.

Extent of resection (EoR) has been established as a prognostic factor for survival in both low- and high-grade diffuse gliomas.^9–13^ However, this may not necessarily apply to each molecular subtype of diffuse glioma. Theoretically, molecular characteristics can influence survival in two ways: either directly through intracellular pathways inducing relatively indolent or aggressive tumor behavior, or indirectly via EoR (Figure 1). It is unclear to what extent surgical resectability of diffuse gliomas is influenced by their molecular profile, for instance, through preferential anatomical locations. As EoR is one of the very few prognostic factors influenceable by physicians, it is important to understand the relations between molecular subtype, anatomical location, and EoR. Research on anatomical location and EoR shows better resectability in frontal tumors.^14^ Small and superficially located tumors in noneloquent areas are more likely to be extensively resected, whereas surgical options in deep seated gliomas in the basal ganglia are frequently limited to biopsy to preserve neurological function. In order to address the relations with molecular subtype, we performed a systematic review of literature, aiming to answer the following questions:

**Figure 1.**
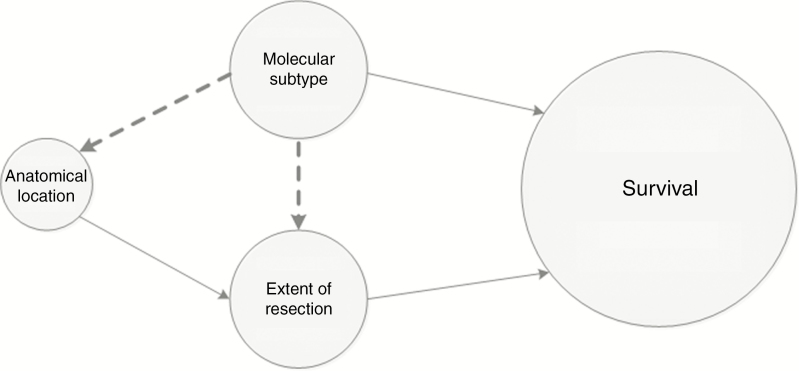
Potential interrelationships between molecular subtype, anatomical location, and extent of resection that can affect survival. The closed lines represent relationships that have been described in previous literature. The dashed lines represent the potential associations that are studied in this review.

- Research question 1: Is anatomical location of diffuse glioma (according to the WHO 2016 classification) correlated with molecular subtype?
- Research question 2: Is EoR of diffuse glioma (according to the WHO 2016 classification) correlated with molecular subtype?

## Materials and Methods

A systematic review was performed in accordance with the Preferred Reporting Items for Systematic Reviews and Meta-Analysis (PRISMA).^15^ A comprehensive electronic search was conducted through PubMed in November 2017. The search comprised terms indicating glioma or a subtype hereof, terms regarding histological or molecular classification and terms either related to anatomical localization or EoR (Supplementary Table S1, Supplementary Material 1).

### Study Eligibility

Studies eligible for inclusion in this review were observational or interventional and prospective as well as retrospective. Only manuscripts written in English, German, French, or Dutch were considered for inclusion.

### Study Selection

Titles and abstracts were screened to identify potentially eligible studies. Full-text articles were selected based on the following inclusion criteria: (a) the patients involved were diagnosed with a grade two, three, or four diffuse glioma based on histopathological examination, and (b) molecular glioma subtype (according to WHO 2016 criteria,^1^ or derivable into WHO 2016 classification) as well as anatomical localization and/or EoR were reported. Only data of adult patients (aged ≥ 18 years) were included in the review. Reviews and case studies were excluded. References of included studies were screened for additional studies eligible for inclusion. All steps of study selection and data extraction were performed by a junior researcher (B.d.L.) and reviewed by K.v.B. Discussion regarding inclusion or data extraction was solved in consensus meetings.

### Quality Assessment

The methodological quality and risk of bias in individual studies were assessed with signaling questions adopted from the Quality Assessment Tool for Observational Cohort and Cross-Sectional Studies^16^ and additional signaling questions considered relevant to this review were formulated by the authors (Supplementary Table S2, Supplementary Material 1). Studies were allocated points based on the extent to which they satisfied the quality criteria and a total score was provided for each study. Quality items weighed unevenly in the total score, taking into consideration the amount of bias they could introduce (Supplementary Material 2). For both research questions, rankings were made per molecular marker. Taking into account the epidemiological questions we aimed to answer, adherence to a systematic approach was particularly important. We decided to apply a threshold for quality score and exclude studies considered to be of poor methodological quality in order to minimize bias. The preferred cutoff was set at nine out of sixteen points. To assess the effect of using this cutoff, we performed an explorative sensitivity analysis with lower (i.e., more liberal) thresholds.

### Data Extraction

Information on selection of participants, patient characteristics, molecular markers, anatomical localization and/or EoR, statistics, and outcomes was obtained from each study when available (Supplementary Table S3, Supplementary Material 1) and entered into a predefined electronic data extraction form.

### Statistical Analysis, Meta-Analysis, and Data Synthesis

In order to unravel potential correlations between molecular subtype and anatomical location or EoR, we re-analyzed all raw data provided by included studies using chi-squared tests in IBM SPSS Statistics 24. Studies using similar outcome measures were included in our systematic study comprising a visual overview of individual study findings and meta-analysis. For the latter, we also applied chi-squared tests. Given the finding that included studies were heterogeneous in definitions of tumor location, a meta-analysis for research question 1 could only be performed after dichotomization of the outcome measures into frontal versus non-rontal, temporal versus nontemporal, parietal versus nonparietal, and “eloquent” versus “non-eloquent” localization. Data on EoR (research question 2) were too heterogeneous in presentation to allow for formal meta-analysis. Study findings that could not be included in the systematic study were synthesized narratively. For both research questions, included studies were categorized by the molecular markers investigated and findings were described correspondingly.

## Results

### Study Selection and Quality Assessment

**Figure 2.**
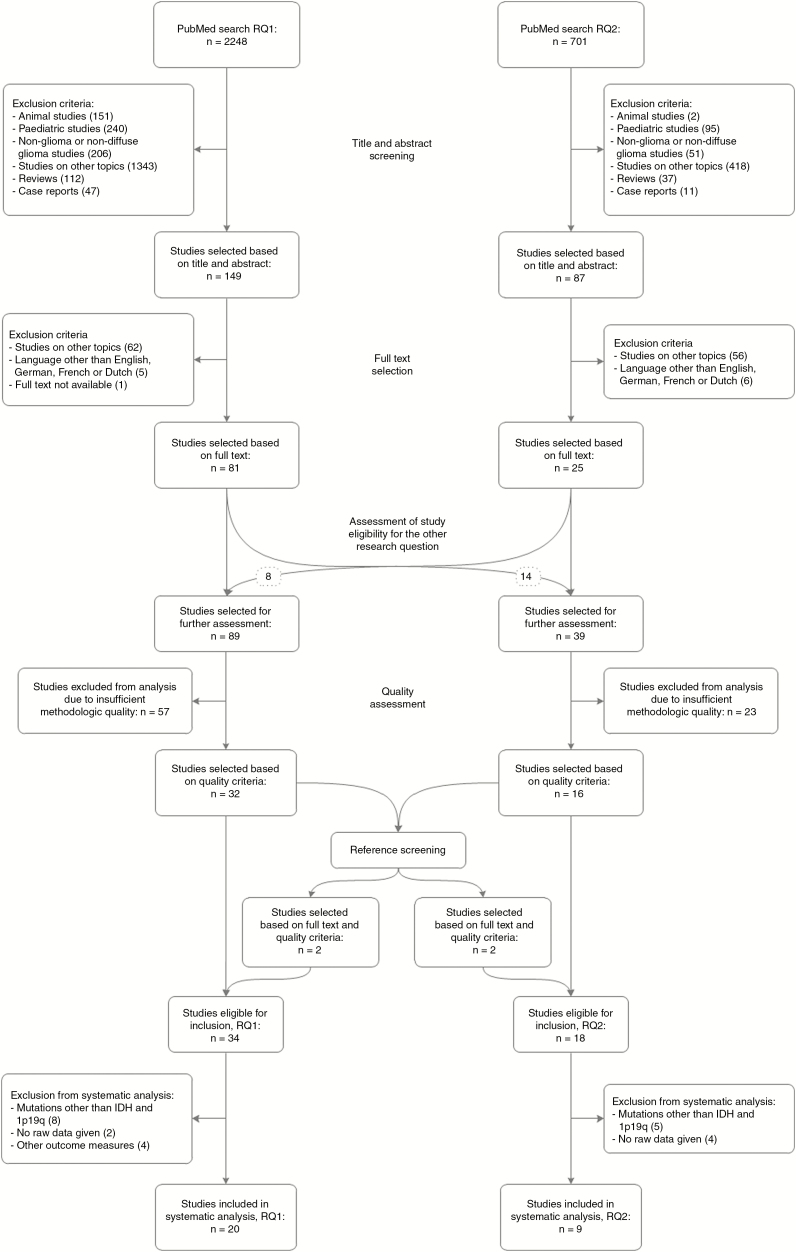
Flowchart of study selection. RQ = research question.

Literature searches for research question 1 and 2 yielded 2248 and 701 studies, respectively. After article screening and selection, 89 and 39 studies were selected to undergo further assessment. Hereof, 34 and 18 studies were considered eligible for inclusion based on quality criteria (Supplementary Tables S4 and S5, Supplementary Material 3). Although the preferred cutoff was set at nine points, we decided to lower it for molecular markers on which limited data were available, in order to optimize the balance between quality standards and amount of data. The quality score threshold for research question 1 was set at 9 for IDH mutation and 1p/19q co-deletion and 8 for other mutations. Lowering the threshold allowed for inclusion of fifteen instead of eight studies on other mutations and multiple studies per mutation. For research question 2, the threshold was set at 9 for IDH mutation and 8 for 1p/19q co-deletion and other mutations. Lowering the threshold raised the number of studies included for 1p/19q co-deletion from four to six and it allowed for inclusion of eight instead of five studies on other mutations and multiple studies per mutation. Excluded studies were considered to be more prone to bias mostly due to unclear or inadequate selection criteria, unclear or inadequate determination of mutational status, anatomical localization and/or EoR, and small sample size. These excluded data corresponded with our results, which implies that excluding them entails minimal risk of introducing selection bias. Data regarding IDH mutation and 1p/19q co-deletion were sufficient to enable systematic study, data concerning other mutations are described in Supplementary Material 4. Ultimately, 20 and 9 studies were included in the systematic analysis (Figure 2). Studies on IDH mutation and 1p/19q co-deletion that lacked raw data or applied other outcome measures are described in the discussion.

### Research Question 1: Mutation Status and Anatomical Location

A total of 20 studies were included in the systematic analysis of mutation status in relation to anatomical location. All were cohort studies. Data were collected retrospectively in eighteen studies and prospectively in two studies. Eleven studies focused on imaging, five on clinical or demographic characteristics and four on survival. The median number of participants in these studies was 147 (range: 25–406) and the median quality score was 10 out of 16 (range: 9–14).

#### IDH mutation

**Figure 3.**
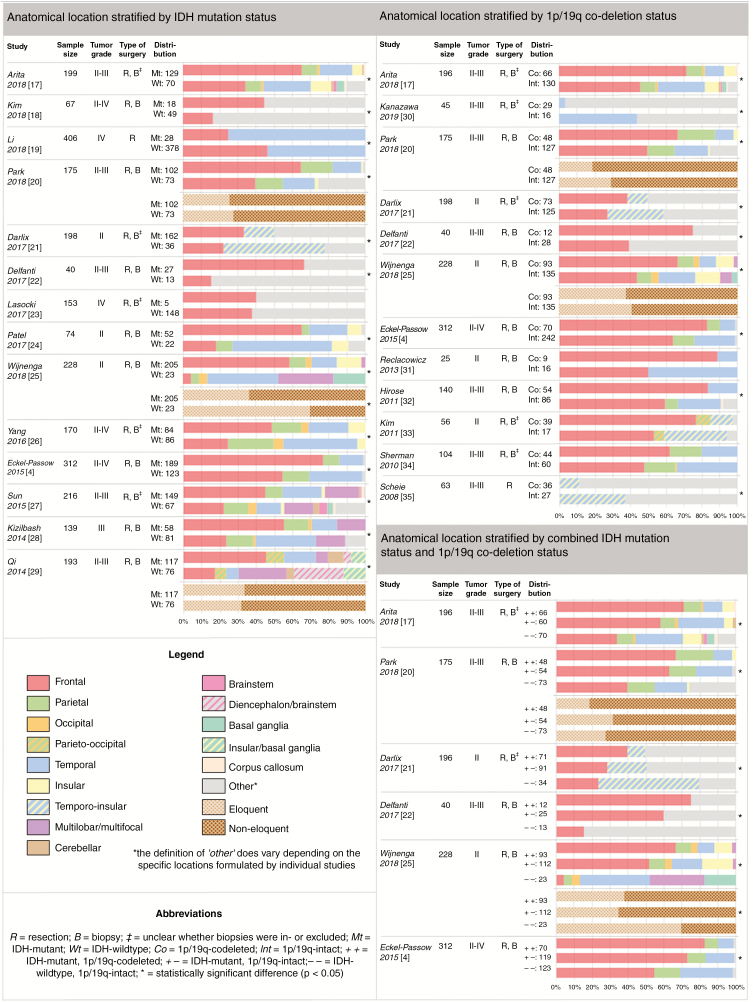
Anatomical location stratified by IDH mutation status, 1p/19q co-deletion status, and combined mutational status, presented per individual study.

Thirteen out of fourteen studies on IDH mutation show a statistically significant difference in the anatomical distribution of IDH-mutant versus IDH-wildtype diffuse gliomas. In all but one study, IDH-mutant tumors were more frequently localized in the frontal lobe compared with IDH-wildtype tumors.^4,17–29^ Regarding eloquent localization, one of three studies reports that IDH-mutant gliomas were significantly more often located in noneloquent regions compared with IDH-wildtype tumors.^25^ The other two studies did not reach statistical significance (Figure 3).^20,29^

#### 1p/19q co-deletion

Nine out of twelve studies on 1p/19q co-deletion demonstrated a statistically significant difference in the anatomical distribution of codeleted versus 1p/19q-intact gliomas. Findings of all studies that specified frontal or temporal localization, respectively, showed that codeleted gliomas were more frequently located in the frontal lobe and less often in the temporal lobe compared with 1p/19q-intact tumors.^4,17,20–22,25,30–35^ Two studies studied eloquent localization in relation to 1p/19q co-deletion status; analysis of their findings revealed no significant correlation (Figure 3).^20,25^

#### Combined mutational status

All six studies that stratified diffuse gliomas by combined IDH mutation and 1p/19q co-deletion status found statistically significant differences in the anatomical distribution of these molecular subgroups. Findings of all studies show that IDH-mutant tumors were significantly more frequently located in the frontal lobe and less often in the temporal lobe compared with IDH-wildtype gliomas. Within the IDH-mutant group, 1p/19q-codeleted tumors were associated with more frequent frontal and less frequent temporal localization compared with 1p/19q-intact tumors.^4,17,20–22,25^ One of two studies on eloquent localization in relation to combined mutational status reveals a significant correlation,^25^ indicating that IDH-wildtype gliomas are more often located in eloquent regions than IDH-mutant tumors regardless of 1p/19q co-deletion status (Figure 3).

#### Meta-analysis

As described in Methods, we performed a meta-analysis with a dichotomous classification of outcomes based on frontal, temporal, parietal, and eloquent localization.

**Figure 4.**
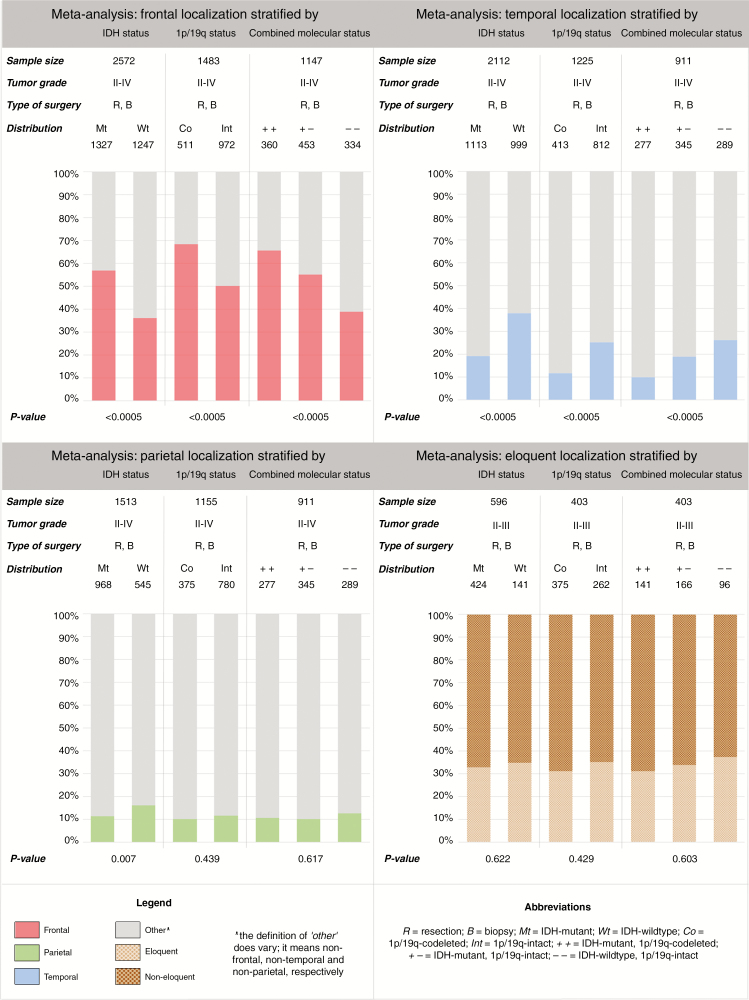
Meta-analysis of frontal, temporal, parietal, and eloquent localization stratified by IDH mutation status, 1p/19q co-deletion status, and combined mutational status.

Our meta-analysis showed statistically significant differences in frontal and temporal localization of diffuse gliomas stratified by IDH mutation, 1p/19q co-deletion, and combined mutational status, respectively. IDH-mutant tumors were more frequently located in the frontal lobe and less often in the temporal lobe compared to IDH-wildtype gliomas (*P* < .0005). Within the IDH-mutant group, 1p/19 co-deletion was associated with more frequent frontal and less frequent temporal locations compared with 19/1q-intact tumors. IDH-wildtype status was further associated with parietal tumor location (*P* = .007). A correlation between 1p/19q co-deletion status or combined mutational status and parietal localization was not found (*P* = .439 and *P* = .617, respectively). IDH mutation, 1p/19q co-deletion, and combined mutational status did not affect eloquent localization of gliomas (*P* = .622, *P* = .429, and *P* = .603, respectively) (Figure 4).

### Research Question 2: Mutation Status and EoR

In the systematic analysis of mutation status in relation to EoR, nine studies were included. All were cohort studies. Data were collected retrospectively in eight studies and prospectively in one study. Four studies primarily focused on clinical or demographic characteristics, three on survival and two were imaging studies. The median number of participants per study was 141 (range: 22–709) and the median quality score was 9 out of 16 (range: 8–13). No formal meta-analysis was possible.

#### IDH mutation

**Figure 5.**
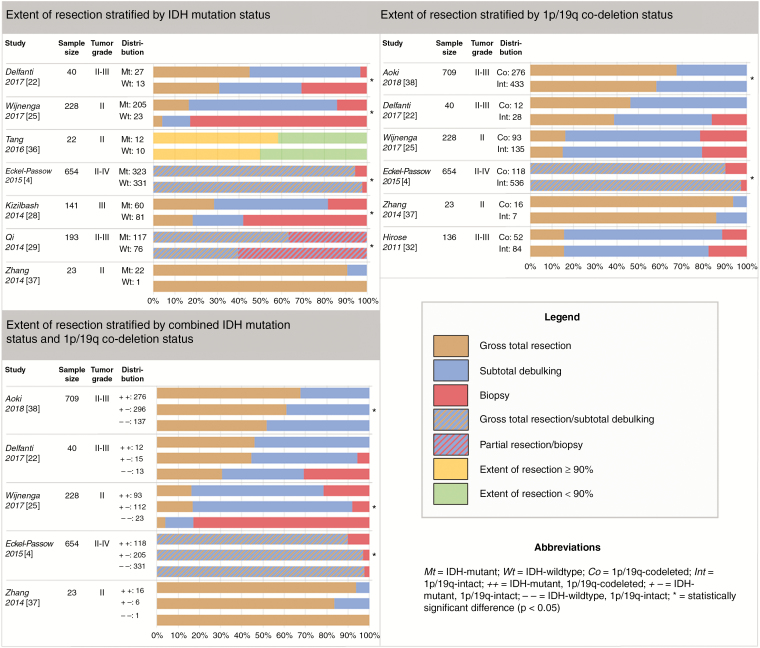
Extent of resection stratified by IDH mutation status, 1p/19q co-deletion status, and combined mutational status, presented per individual study.

Five out of seven studies on IDH mutation showed a statistically significant difference in the EoR of IDH-mutant versus IDH-wildtype diffuse gliomas.^4,22,25,28,29,36,37^ In four studies, IDH-mutant tumors showed better resectability and the fifth study indicated greater EoR in IDH-wildtype tumors (Figure 5).^4,22,25,28,29^

#### 1p/19q co-deletion

Two out of six studies on 1p/19q co-deletion demonstrated a statistically significant difference in the EoR of codeleted versus 1p/19q-intact gliomas.^4,22,25,32,37,38^ One study indicates that 1p/19q-intact tumors are more extensively resected, the other demonstrates better resectability of codeleted tumors.^4,38^ The studies without significant results mostly show trends toward the latter (Figure 5).^22,32,37^

#### Combined mutational status

Three of five studies that stratified diffuse gliomas by combined IDH mutation and 1p/19q co-deletion status found statistically significant differences in the EoR of these molecular subgroups.^4,22,25,37,38^ Two studies found better resectability of IDH-mutant gliomas compared with IDH-wildtype gliomas.^25,38^ One of these studies also found that within IDH-mutant gliomas, EoR was greater in 1p/19q-codeleted tumors compared with 1p/19q-intact tumors.^38^ Results of the third study demonstrate greater EoR of both IDH-wildtype and IDH-mutant, 1p/19q-intact gliomas compared to IDH-mutant, codeleted tumors (Figure 5).^4^

## Discussion

### Main Findings

This systematic review and meta-analysis demonstrates that IDH-mutant gliomas are more frequently located in the frontal lobe and less often in the temporal lobe than IDH-wildtype, 1p/19q-intact gliomas. Within the group of IDH-mutant tumors, 1p/19q-codeleted tumors occur more often in the frontal lobes, and less often in the temporal lobe, than 1p/19q-intact tumors.

Data on the correlation between molecular status and EoR of gliomas are less consistent, with no possibility for meta-analysis. Data of individual studies suggest that in patients suffering from IDH-mutant gliomas (regardless of their 1p/19q status) gross total resections are more frequently achieved and these patients are less likely to undergo a biopsy compared with patients with IDH-wildtype, 1p/19q-intact gliomas. Data on other mutations, such as TERT and TP53, were insufficient to describe a correlation between anatomical location or EoR.

### EoR, Molecular Subtype, and Anatomical Location

Our findings of correlations between molecular subtype and EoR as well as between molecular subtype and anatomical location suggest that EoR may be influenced by molecular subtype, possibly through preferential anatomical locations of molecularly defined diffuse glioma (Figure 1). These associations are further supported by several studies that are not included in the formal review due to absence of raw data or application of other outcome measures.

Regarding IDH mutation, these studies too report wildtype gliomas to be more frequently located in the temporal lobe,^30^ insula,^39^ brainstem, and other midline localizations.^18,27,40,41^ IDH wildtype status is also correlated with the presence of multifocal tumors.^40^ Furthermore, an association between presence of IDH mutation and surface localization was found.^42^ In accordance with our results, EoR was reported to be greater in IDH-mutant gliomas.^24,43^ A single study involving 200 patients describes not finding a correlation between IDH mutation status and EoR.^44^

Concerning 1p/19q co-deletion, studies report high incidences of codeleted gliomas in the right frontal lobe and anterior insula^45^ and in frontal location,^46^ as we found. A positive association between presence of a co-deletion and surface localization^42^ is reported, as well as absence of such a correlation.^30^ Regarding the relation between 1p/19q co-deletion and EoR, results are ambivalent as one study reports lower EoR in codeleted gliomas^44^ and another describes not finding an association.^47^

Of course, there may be factors other than localization and molecular subtype that also have an influence on EoR. The hypothesis of the relation between molecular subtype and EoR being confounded by anatomical location is endorsed by our own results as well as other studies’ findings. Our results demonstrate that IDH-mutant gliomas, especially those which are also 1p/19q-codeleted, are more frequently located in the frontal lobe, where higher resection percentages are achieved,^6^ and less often in temporal areas. Moreover, they show that these tumors are more frequently completely resected and less often biopsied. Only three studies have focused on the relation between location and EoR. Analysis performed by Beiko et al.^14^ demonstrates that frontal location and IDH mutation are independent prognostic factors for complete resection of high-grade astrocytoma (*P* = .01 and .03, respectively). Wijnenga et al.^25^ report that insular and eloquent localization are associated with greater postoperative tumor volume in diffuse low-grade glioma (*P* < .0001 for both factors), as well as increasing age and preoperative tumor volume (*P* = .002 and *P* < .0001, respectively). Moreover, they state that corrected for these factors, molecular subtype did not correlate with postoperative tumor volume. This supports the idea that the relation between molecular subtype and EoR is mediated by anatomical location. More research in adequate-sized (prospective) cohorts is needed to elucidate whether location—and possibly other known prognostic factors such as age—fully explains the association between molecular subtype and EoR, or whether other mechanisms and factors mediate this relationship.

### Prognostic Value of EoR and Molecular Subtype

Prognostic value of molecular subtype and EoR has been well established. However, as we have demonstrated that a correlation between the two exists, a confounding effect may be in play. The key question is whether EoR holds up as a prognostic factor for survival after correction for contemporary molecular subtyping and subsequent glioma classification. Several studies have performed multivariable analysis including both EoR and molecular markers. Metellus et al.^39^ found that in low-grade gliomas tumor location, tumor diameter on MRI, EoR, and IDH mutation were prognostic factors in univariable analysis (*P* = .025, .038, .039, and .00002, respectively), yet in multivariable analysis only IDH mutation held up as an independent prognostic factor (*P* = .001). Likewise, Wijnenga et al.^25^ found that in low-grade gliomas eloquent tumor localization, resection percentage, and molecular diagnosis are prognostic factors univariably (*P* = .004, *P* < .0001, and *P* < .0001, respectively) and in multivariable analysis only molecular diagnosis was an independent prognostic factor (*P* = .0001). Their results also do not demonstrate categorized EoR or postoperative tumor volume to be statistically significant prognostic factors for overall survival in IDH-mutant low-grade gliomas, regardless of their 1p/19q-status. This analysis was not feasible in the IDH-wildtype group due to small sample size. Findings of above-mentioned studies suggest that the prognostic value of EoR is better explained by molecular diagnosis whenever correction for this factor takes place. Beiko et al.^14^ studied high-grade astrocytomas and their univariable analysis demonstrates that in IDH-wildtype tumors, EoR was not a prognostic factor, though postoperative tumor volume was (*P* = .021). However, this did not hold up after correction for preoperative enhancement and age in the multivariable analysis. In the IDH-mutant group preoperative volume, postoperative volume and EoR were indicated as prognostic markers univariably (*P* = .054, .001, and .03, respectively), but only preoperative and postoperative volume remained significant in the multivariable analysis (*P* = .01 and *P* < .001, respectively). By contrast, analysis performed by Patel et al.^24^ in low-grade gliomas demonstrates that EoR is a prognostic factor in IDH-wildtype tumors, but not in the IDH-mutant group (*P* = .003 and .48, respectively). Current data are too heterogeneous and insufficient to draw any conclusions concerning relative prognostic value of EoR and molecular subtype.

Studies on EoR of diffuse glioma should be stratified by molecular subgroups as outlined by the WHO 2016 classification. Until more of these studies become available, no clear recommendations can be given regarding surgical management for different molecular subgroups; until then, current guidelines favoring maximum safe resection should be considered as standard of care.

### Strengths and Limitations

The main conceptual strength of this review/meta-analysis is the systematic study of the interrelationships between tumor type, location, and EoR in the context of current-era, molecular marker-based classification of gliomas (WHO 2016). Methodological strengths of this review include its systematic set-up and search, and the standardized assessment of methodological quality of included studies. The review’s limitations are secondary to the limitations of the underlying studies, including potential selection bias due to mere inclusion of resected and biopsied gliomas—without regard for patients who are on a watchful waiting-strategy. Also, we did not analyze the degree of potential reporting and publication bias. Furthermore, the majority of included studies classified anatomical location by brain lobes. This is probably not sufficient when it comes to correlating anatomical location of brain tumors to their EoR. Location in or adjacent to eloquent regions is more likely to influence EoR. Only a limited number of studies looked into eloquence of tumor location, with heterogeneous and sparse definitions of eloquent location.

## Conclusions

Genetic profile of diffuse cerebral glioma influences their anatomical location. Available evidence strongly suggests that it also affects tumor resectability.

### Implications for Future Research

Further research should focus on influence of molecular markers on occurrence of diffuse glioma in eloquent brain regions and analyze this in relation to extent of resection. Furthermore, multivariable regression analyses taking into account molecular subtype, extent of resection, and anatomical as well as eloquent localization should be performed in a diffuse glioma sample, as this would enable elucidation of the relative prognostic value of these factors. Future studies concerning extent of resection should stratify their results by molecular subgroups as outlined by the WHO 2016 classification. These lines of research should finally answer the question whether the surgical strategy for a diffuse glioma is dependent on the molecular subtype. If this is the case, then preoperative prediction of molecular subtype with advanced imaging becomes increasingly important.

Tumor localization should be taken into account as a possible confounder in comparative prognostic research regarding diffuse cerebral glioma. Lastly, there is need for research into the relations between anatomical location, MRI characteristics, and extent of resection with the aim of finding out whether MRI characteristics are predictive of EoR and to what degree relative to anatomical location.

### Implications for Practice

Knowledge of prognostic value of molecular markers, extent of resection, and anatomical as well as eloquent localization will enable more specific formulation of recommendations for surgical management of diffuse glioma. These should ultimately be stratified by molecular subgroups as outlined by the WHO 2016 classification. This would further launch oncological neurosurgery into the era of precision medicine.

## Supplementary Material

vdz032_suppl_Supplementary_material
