# Supplementary material for: Interrelationships between molecular subtype, anatomical location, and extent of resection in diffuse glioma: a systematic review and meta-analysis
Source: Neurooncol Adv. 2019 Oct 1;1(1):vdz032. doi: 10.1093/noajnl/vdz032 (PMC7212862; doi:10.1093/noajnl/vdz032)
Supplement: vdz032_suppl_Supplementary_material [file vdz032_suppl_supplementary_material.pdf]

Supplement 1 – Search terms, quality assessment and data extraction tables

Table S1. Search terms

**Glioma**	(glioma OR gliomas OR glioblastoma OR glioblastomas OR astrocytoma OR astrocytomas OR astrocytic OR oligodendrogli* OR oligoastrocyt*).tiab
AND	
**Classification**	(histology OR histologic OR molecular OR genetic OR mutation OR mutant OR mutated OR co-delet* OR pathophysiologic* OR methyl* OR MGMT OR amplifi* OR EGFR).tiab
AND	
**Localization** *(For research question 1 only)*	(location* OR localization* OR lobe* OR radiographic* OR radiologic*) .tiab
AND	
**Extent of resection** *(For research question 2 only)*	(“extent of resection” OR “extent of surgery” OR “gross total resection” OR “complete resection” OR “complete surgical resection” OR “subtotal resection” OR “subtotal surgical resection” OR “incomplete resection” OR “incomplete surgical resection” OR “partial resection” OR “partial surgical resection” OR “type of surgery” OR debulking).tiab

Table S2. Quality assessment signaling questions and score

Signaling question	Maximum number of points
Was the study population clearly specified and defined?	2
Was the participation rate of eligible persons at least 50%?	1
Was the study performed in a population of at least 50 participants?	1
Were participants selected consecutively (uniform application of inclusion and exclusion criteria and participants from the same or similar populations)?	2
Was mutational status clearly defined, valid, reliable, and implemented consistently across all study participants?	2
Was anatomical localization (question 1) or EoR / type of surgery (question 2) clearly defined, valid, reliable, and implemented consistently across all study participants?	3
Were the outcome assessors blinded to the mutational status of the examined tumors?	1
Does the study sample match the review domain in its full breadth (minimization of selection bias)?	4
Maximum number of points	16

Table S3. Definition of data extraction items

**Study**	first author and year of publication
**Selection of participants**	duration and years of sample collection, inclusion and exclusion criteria
**Patient characteristics**	number of participants, gender distribution, age (either mean or median, and range), type of glioma (histology, grade and distribution hereof)
**Molecular markers**	molecular markers investigated
**Anatomical localization (research question 1)**	anatomical localizations reported
**Extent of resection (research question 2)**	threshold(s) applied for EoR or types of surgery compared
**Statistics**	statistical tests performed
**Outcomes**	conclusions regarding relation of molecular markers to<br>- localization (research question 1) and/or<br>- EoR or type of surgery (research question 2)<br>and reported frequencies of occurrence regarding these relations

Supplement 2 – Quality assessment scoring system

Points / Quality item	0	1	2	3	4
Description of study population:<br>1. Age<br>2. Tumor type (histological or molecular)<br>3. Tumor grade	Two or more characteristics are missing	One of three characteristics is missing	All three characteristics are described	x	x
Participation rate of eligible persons	< 50% or unknown	≥ 50%	x	x	x
Sample size	< 50 participants	≥ 50 participants	x	x	x
Consecutive selection of participants:<br>1. Time period<br>2. In- and/or exclusion criteria<br>3. Number of persons in- or excluded per criterion	Items other than no. 3 are missing	Only item 3 is missing	All three items are described	x	x
Determination of mutation status	Unknown	Fluorescence in situ hybridiza-tion or immuno-histochemistry	Polymerase chain reaction / next generation sequencing	x	x
Determination of anatomical location / extent of resection	Unknown	CT, 'radiological' or 'imaging' (unspecified whether this indicates MRI)	MRI (anatomical location: without defining anatomical locations<br>EoR: application of other sequences than described in the adjacent column or without specifying applied sequences)	MRI (anatomical location: with definitions of allocated anatomical locations<br>EoR: application of T2 or FLAIR sequences in low-grade and T1 sequences with contrast in high-grade glioma)	x
Blinding	Not applied or unknown	Applied	x	x	x
Minimization of selection bias	A maximum of 4 points can be allocated for this quality item. The following features are grounds for deduction of points.<br>- Selection based on tumor grade (-1 point)<br>- Selection based on tumor histology, mutation status, anatomical location or treatment (-2 points per selection criterion)<br>- No mention of selection (in- or exclusion) criteria (-4 points)				

Supplement 3 – Quality assessment

Table S4. Assessment of methodological quality and risk of bias of studies included for Research Question 1

	Description of study population	Participation rate ≥ 50%	No. of participants ≥ 50	Consecutive inclusion of participants	Reliable determination of mutational status	Reliable determination of localization	Blinding	Minimization of selection bias	Quality score
Arita 2018[1]	+	-	+	-	+	+	-	+	10 (IDH, 1p/19q, TERT, MGMT)
Izquierdo 2019[2]	+	-	+	=	+	+	-	-	8 (TERT)
Kanazawa 2019[3]	+	-	-	=	+	+	+	+	11 (IDH, 1p/19q, MGMT)
Kim 2018[4]	+	-	+	=	+	+	+	+	13 (IDH, TERT, MGMT)
Li 2018[5]	+	-	+	=	+	+	+	-	9 (IDH, MGMT)
Park 2018[6]	+	-	+	+	+	+	+	+	13 (IDH)
	+	-	+	+	=	+	+	+	12 (1p/19q)
Villanueva-Meyer 2018[7]	=	-	+	=	=	+	+	+	10 (IDH)
Wang 2018[8]	+	-	+	=	=	+	+	=	9 (1p/19q)
Williams 2018[9]	+	-	+	+	+	-	-	+	10 (MGMT)
	+	-	+	+	+	-	-	=	8 (TERT)
Akyerli 2018[10]	+	-	+	-	+	-	-	+	8 (TERT)
Darlix 2017[11]	+	+	+	+	+	+	+	+	14 (IDH, 1p/19q)
Delfanti 2017[12]	+	+	-	+	=	+	+	+	12 (IDH, 1p/19q)
Lasocki 2017[13]	=	+	+	+	=	+	-	=	9 (IDH)
Pai 2017[14]	+	+	+	+	=	-	-	=	9 (1p/19q)
Patel 2018[15]	+	-	+	+	=	-	-	+	9 (IDH)
Wijnenga 2018[16]	+	+	+	+	+	-	-	=	10 (IDH, 1p/19q)
Yang 2016[17]	+	-	+	=	+	+	-	=	10 (IDH)
Yuan 2016[18]	=	-	+	=	=	+	-	=	8 (MGMT, P53, EGFR)

Zhang *2016*[19]	+	-	+	=	+	-	-	+	9 (IDH)
	+	-	+	=	=	-	-	+	8 (EGFR)
Eckel-Passow *2015*[20]	+	-	+	=	+	=	-	+	11 (IDH, TERT)
	+	-	+	=	=	-	-	+	9 (1p/19q)
Sun *2015*[21]	+	-	+	=	=	+	+	+	11 (IDH, TERT)
Kizilbash *2014*[22]	=	+	+	+	+	+	-	-	9 (IDH)
Nishiyama *2014*[23]	+	-	+	=	+	+	-	+	11 (IDH, 1p/19q)
Qi *2014*[24]	+	-	+	=	+	+	+	=	10 (IDH)
Reclacowicz *2013*[25]	+	+	-	+	=	-	-	+	9 (1p/19q)
Carrillo *2012*[26]	+	-	+	-	+	+	+	-	8 (MGMT)
Singh *2012*[27]	+	-	-	+	+	-	-	=	8 (MGMT)
Hirose *2011*[28]	+	-	+	=	+	-	-	+	9 (1p/19q)
Kim *2011*[29]	+	-	+	=	=	+	+	=	9 (1p/19q)
Drabycz *2010*[30]	+	+	+	+	+	+	+	+	14 (MGMT)
Metellus *2010*[31]	+	-	-	=	+	+	+	=	10 (IDH)
Sherman *2010*[32]	=	+	+	+	=	+	+	=	10 (1p/19q)
Scheie *2008*[33]	+	+	+	+	+	+	-	=	11 (1p/19q)
Mut *2007*[34]	+	-	+	=	=	+	+	+	11 (P53)

Table S5. Assessment of methodological quality and risk of bias of studies included for Research Question 2

	Description of study population	Participation rate ≥ 50%	No. of participants ≥ 50	Consecutive inclusion of participants	Reliable determination of mutational status	Reliable determination of extent of resection	Blinding	Minimization of selection bias	Quality score
Gessler 2019[35]	+	+	+	+	+	+	+	=	13 (MGMT)
Aoki 2018[36]	+	-	+	-	+	-	-	+	8 (IDH, 1p/19q)
Delfanti 2017[12]	+	-	+	+	=	-	-	+	9 (IDH, 1p/19q)
Eseonu 2017[37]	=	+	-	=	-	+	+	=	8 (1p/19q)
Patel 2018[15]	+	-	+	+	=	+	+	+	13 (IDH)
Wijnenga 2018[16]	+	+	+	+	+	+	-	=	13 (IDH, 1p/19q)
Yan 2017[38]	+	-	-	-	+	+	-	=	8 (MGMT)
Jungk 2016[39]	+	+	-	+	=	+	-	=	10 (IDH)
Tang 2016[40]	+	-	-	=	+	+	-	=	9 (IDH)
Cordier 2015[41]	+	+	+	=	+	+	-	=	12 (IDH, 1p/19q)
	+	+	+	=	=	+	-	=	11 (P53)
Eckel-Passow 2015[20]	+	-	+	=	=	-	-	+	9 (IDH, 1p/19q, TERT)
Aihara 2014[42]	+	+	-	+	+	-	-	=	9 (MGMT)
Kizilbash 2014[22]	=	+	+	+	+	+	-	-	9 (IDH)
Qi 2014[24]	+	-	+	=	+	+	+	=	10 (IDH)
Zhang 2014[43]	+	+	-	+	+	-	-	=	9 (IDH, TERT)
	+	+	-	+	=	-	-	=	8 (1p/19q)
Singh 2012[27]	+	-	-	+	+	-	-	=	8 (MGMT)
Hirose 2011[28]	+	-	+	=	+	+	-	+	11 (1p/19q)
Mut 2007[34]	+	-	+	=	=	-	-	+	8 (P53)

Supplement 4 – Data on other mutations

Mutation status and anatomical location

TERT promoter mutation

Multiple studies describe correlations between TERT promoter mutation status and anatomical location. TERT-mutant tumors were more frequently found in the frontal[1,2,4] and parietal lobe[2]. Studies report conflicting correlations between TERT mutation and localization in the temporal[1,2] and insular lobe[2,10]. TERT-wildtype gliomas would more often be located in the brainstem[2], midline[4] and cerebellum[9] compared to TERT-mutant tumors. However, results of several studies demonstrate absence of an association between TERT mutation status and anatomical location[20,21] or midline localization[21].

MGMT promoter methylation

Several studies report associations between MGMT promoter methylation status and anatomical location. A high frequency of MGMT-wildtype tumors in the right medial frontal lobe is described.[18] However, comparative results regarding frontal[1,5] and temporal[3,5] localization of gliomas stratified by MGMT promoter methylation status are ambivalent. Unmethylated tumors have been found more frequently in the basal ganglia[1] and showed surface[3] and multifocal[26] localization more often compared to methylated tumors. In contrast, findings of multiple studies do not suggest a correlation between MGMT methylation and tumor localization.[4,9,26,27,30]

EGFR gene amplification

Studies that performed EGFR analyses report that gliomas displaying EGFR-overexpression were relatively often localized around the left anterior horn of the lateral ventricle[18] and predominantly in the hemispheres without affecting the midline[19].

P53 mutation

One study describes finding high frequencies of P53-mutant tumors in the anterior temporal lobe, left insula and left lentiform nucleus.[18] Another concluded that different degree of P53 expression did not predict preference for lesion location.[34]

Mutation status and EoR

TERT promoter mutation

Findings of studies that performed TERT promoter mutation analyses demonstrate no correlation between mutation status and EoR.[20,43]

MGMT promoter methylation

None of the findings of the included studies suggested a correlation between MGMT promoter methylation status and EoR.[27,35,38,42]

P53 mutation

Studies report absence of an association between P53 mutation status and EoR.[34,41]

References

1. Arita H, Kinoshita M, Kawaguchi A, et al. Lesion location implemented magnetic resonance imaging radiomics for predicting IDH and TERT promoter mutations in grade II/III gliomas. Sci Rep. 2018; 8(1): 11773.
2. Izquierdo C, Barritault M, Poncet D, et al. Radiological Characteristics and Natural History of Adult IDH-Wildtype Astrocytomas with TERT Promoter Mutations. Neurosurgery. 2019; 85(3): E448-E456.
3. Kanazawa T, Fujiwara H, Takahashi H, et al. Imaging scoring systems for preoperative molecular diagnoses of lower-grade gliomas. Neurosurg Rev. 2019; 42(2): 433-441.
4. Kim HS, Kwon MJ, Song JH, Kim ES, Kim HY, Min KW. Clinical implications of TERT promoter mutation on IDH mutation and MGMT promoter methylation in diffuse gliomas. Pathol Res Pract. 2018; 214(6): 881-888.
5. Li HY, Sun CR, He M, Yin LC, Du HG, Zhang JM. Correlation Between Tumor Location and Clinical Properties of Glioblastomas in Frontal and Temporal Lobes. World Neurosurg. 2018; 112: e407-e414.
6. Park YW, Han K, Ahn SS, et al. Prediction of IDH1-Mutation and 1p/19q-Codeletion Status Using Preoperative MR Imaging Phenotypes in Lower Grade Gliomas. AJNR Am J Neuroradiol. 2018; 39(1): 37-42.
7. Villanueva-Meyer JE, Wood MD, Choi BS, et al. MRI Features and IDH Mutational Status of Grade II Diffuse Gliomas: Impact on Diagnosis and Prognosis. AJR Am J Roentgenol. 2018; 210(3): 621-628.
8. Wang K, Wang Y, Fan X, et al. Regional Specificity of 1p/19q co-deletion combined with radiological features for predicting the survival outcomes of anaplastic oligodendroglial tumor patients. J Neurooncol. 2018; 136(3): 523-531.

9. Williams EA, Miller JJ, Tummala SS, et al. TERT Promoter wild-type glioblastomas show distinct clinical features and frequent PI3K pathway mutations. Acta Neuropathol Commun. 2018; 6(1): 106.
10. Akyerli CB, Yüksel Ş, Can Ö, et al. Use of telomerase promoter mutations to mark specific molecular subsets with reciprocal clinical behavior in IDH mutant and IDH wild-type diffuse gliomas. J Neurosurg. 2018; 128(4): 1102-1114.
11. Darlix A, Deverdun J, Menjot de Champfleur N, et al. IDH mutation and 1p19q codeletion distinguish two radiological patterns of diffuse low-grade gliomas. J Neurooncol. 2017; 113(1): 37-45.
12. Delfanti RL, Piccioni DE, Handwerker J, et al. Imaging correlates for the 2016 update on WHO classification of grade II/III gliomas: implications for IDH, 1p/19q and ATRX status. J Neurooncol. 2017; 135(3): 601-609.
13. Lasocki A, Tsui A, Gaillard F, Tacey M, Drummond K, Stuckey S. Reliability of noncontrast-enhancing tumor as a biomarker of IDH1 mutation status in glioblastoma. J Clin Neurosci. 2017; 39: 170-175.
14. Pai T, Epari S, Desai S, et al. Histological spectrum of oligodendroglial tumors: Only a subset shows 1p/19q codeletion. Neurol India. 2017; 65(1): 113-120.
15. Patel T, Bander ED, Venn RA, et al. The Role of Extent of Resection in IDH1 Wild-Type or Mutant Low-Grade Gliomas. Neurosurgery. 2018; 82(6): 808-814.
16. Wijnenga MMJ, French PJ, Dubbink HJ, et al. The impact of surgery in molecularly defined low-grade glioma: an integrated clinical, radiological, and molecular analysis. Neuro Oncol. 2018; 20(1): 103-112.
17. Yang Y, Mao Q, Wang X, et al. An analysis of 170 glioma patients and systematic review to investigate the association between IDH-1 mutations and preoperative glioma-related epilepsy. J Clin Neurosci. 2016; 31: 56-62.

18. Yuan Y, Yunhe M, Xiang W, et al. Mapping genetic factors in high-grade glioma patients. Clin Neurol Neurosurg. 2016; 150: 159-163.
19. Zhang RQ, Shi Z, Chen H, et al. Biomarker-based prognostic stratification of young adult glioblastoma. Oncotarget. 2016; 7(4): 5030-5041.
20. Eckel-Passow JE, Lachance DH, Molinaro AM, et al. Glioma Groups Based on 1p/19q, IDH, and TERT Promoter Mutations in Tumors. N Engl J Med. 2015; 372(26): 2499-2508.
21. Sun ZL, Chan AK, Chen LC, et al. TERT promoter mutated WHO grades II and III gliomas are located preferentially in the frontal lobe and avoid the midline. Int J Clin Exp Pathol. 2015; 8(9): 11485-11494.
22. Kizilbash SH, Giannini C, Voss JS, et al. The impact of concurrent temozolomide with adjuvant radiation and IDH mutation status among patients with anaplastic astrocytoma. J Neurooncol. 2014; 120(1): 85-93.
23. Nishiyama Y, Sasaki H, Nagahisa S, et al. Radiological features of supratentorial gliomas are associated with their genetic aberrations. Neurosurg Rev. 2014; 37(2): 291-299.
24. Qi S, Yu L, Li H, et al. Isocitrate dehydrogenase mutation is associated with tumor location and magnetic resonance imaging characteristics in astrocytic neoplasms. Oncol Lett. 2014; 7(6): 1895-1902.
25. Ręcławowicz D, Stempniewicz M, Biernat W, Limon J, Słoniewski P. Loss of genetic material within 1p and 19q chromosomal arms in low grade gliomas of central nervous system. Folia Neuropathol. 2013; 51(1): 26-32.
26. Carrillo JA, Lai A, Nghiemphu PL, et al. Relationship between tumor enhancement, edema, IDH1 mutational status, MGMT promoter methylation, and survival in glioblastoma. AJNR Am J Neuroradiol. 2012; 33(7): 1349-1355.
27. Singh G, Mallick S, Sharma V, et al. A study of clinico-pathological parameters and O6 -methylguanine DNA methyltransferase (MGMT) promoter methylation status in the prognostication of gliosarcoma. Neuropathol. 2012; 32(5): 534-542.

28. Hirose Y, Sasaki H, Miwa T, et al. Whole genome analysis from microdissected tissue revealed adult supratentorial grade II-III gliomas are divided into clinically relevant subgroups by genetic profile. Neurosurgery. 2011; 69(2): 376-390.
29. Kim JW, Park CK, Park SH, et al. Relationship between radiological characteristics and combined 1p and 19q deletion in World Health Organization grade III oligodendroglial tumours. J Neurol Neurosurg Psychiatry. 2011; 82(2): 224-227.
30. Drabycz S, Roldán G, de Robles P, et al. An analysis of image texture, tumor location, and MGMT promoter methylation in glioblastoma using magnetic resonance imaging. Neuroimage. 2010; 49(2): 1398-1405.
31. Metellus P, Coulibaly B, Colin C, et al. Absence of IDH mutation identifies a novel radiologic and molecular subtype of WHO grade II gliomas with dismal prognosis. Acta Neuropathol. 2010; 120(6): 719-729.
32. Sherman JH, Prevedello DM, Shah L, et al. MR imaging characteristics of oligodendroglial tumors with assessment of 1p/19q deletion status. Acta Neurochir (Wien). 2010; 152(11): 1827-1834.
33. Scheie D, Cvancarova M, Mørk S, et al. Can morphology predict 1p/19q loss in oligodendroglial tumours? Histopathology. 2008; 53(5): 578-587.
34. Mut M, Turba UC, Botella AC, Baskurt E, Lopes MB, Shaffrey ME. Neuroimaging characteristics in subgroup of GBMs with P53 overexpression. J Neuroimaging. 2007; 17(2): 168-174.
35. Gessler F, Bernstock JD, Braczynski A, et al. Surgery for Glioblastoma in Light of Molecular Markers: Impact of Resection and MGMT Promoter Methylation in Newly Diagnosed IDH-1 Wild-type Glioblastomas. Neurosurgery. 2019; 84(1): 190-197.
36. Aoki K, Nakamura H, Suzuki H, et al. Prognostic relevance of genetic alterations in diffuse lower-grade gliomas. Neuro Oncol. 2018; 20(1): 66-77.

37. Eseonu CI, ReFaey K, Garcia O, Raghuraman G, Quinones-Hinojosa A. Volumetric Analysis of Extent of Resection, Survival, and Surgical Outcomes for Insular Gliomas. World Neurosurg. 2017; 103: 265-274.
38. Yan JL, van der Hoorn A, Larkin TJ, Boonzaier NR, Matys T, Price SJ. Extent of resection of peritumoral diffusion tensor imaging-detected abnormality as a predictor of survival in adult glioblastoma patients. J Neurosurg. 2017; 126(1): 234-241.
39. Jungk C, Scherer M, Mock A, et al. Prognostic value of the extent of resection in supratentorial WHO grade II astrocytomas stratified for IDH1 mutation status: a single-center volumetric analysis. J Neurooncol. 2016; 129(2): 319-328.
40. Tang C, Zhang ZY, Chen LC, et al. Subgroup characteristics of insular low-grade glioma based on clinical and molecular analysis of 42 cases. J Neurooncol. 2016; 126(3): 499-507.
41. Cordier D, Gozé C, Schädelin S, Rigau V, Mariani L, Duffau H. A better surgical resectability of WHO grade II gliomas is independent of favorable molecular markers. J Neurooncol. 2015; 121(1): 185-193.
42. Aihara K, Mukasa A, Gotoh K, et al. H3F3A K27M mutations in thalamic gliomas from young adult patients. Neuro Oncol. 2014; 16(1): 140-146.
43. Zhang ZY, Chan AK, Ng HK, et al. Surgically treated incidentally discovered low-grade gliomas are mostly IDH mutated and 1p19q co-deleted with favorable prognosis. Int J Clin Exp Pathol. 2014; 7(12): 8627-8636.
